# Supplementary material for: Comparative genomics provides new insights into the diversity, physiology, and sexuality of the only industrially exploited tremellomycete: Phaffia rhodozyma
Source: BMC Genomics. 2016 Nov 9;17:901. doi: 10.1186/s12864-016-3244-7 (PMC5103461; doi:10.1186/s12864-016-3244-7)
Supplement: Additional file 6: — List of orphan genes with links to PFAM (related to Additional file 1: Table S1). (ZIP 1428 kb) [file 12864_2016_3244_MOESM6_ESM.zip › PFAM_UNIQUE/G05470_P.html]

#                                                                            --- full sequence --- -------------- this domain -------------   hmm coord   ali coord   env coord
  
# target name        accession   tlen query name           accession   qlen   E-value  score  bias   #  of  c-Evalue  i-Evalue  score  bias  from    to  from    to  from    to  acc description of target
  
#------------------- ---------- ----- -------------------- ---------- ----- --------- ------ ----- --- --- --------- --------- ------ ----- ----- ----- ----- ----- ----- ----- ---- ---------------------
  
G05470\_P             -            333 DUF3460              PF11943.3     60       0.2   10.5   2.6   1   3       0.7   4.2e+03   -3.4   0.0    18    33    98   113    96   114 0.82 - Protein of unknown function (DUF3460)  
G05470\_P             -            333 DUF3460              PF11943.3     60       0.2   10.5   2.6   2   3      0.02   1.2e+02    1.6   0.1    21    34   188   201   187   211 0.87 - Protein of unknown function (DUF3460)  
G05470\_P             -            333 DUF3460              PF11943.3     60       0.2   10.5   2.6   3   3   0.00018       1.1    8.1   0.1     7    40   294   327   289   330 0.88 - Protein of unknown function (DUF3460)
